# An Overview of Ascariasis Involvement in Gallbladder Disease: A Systematic Review of Case Reports

**DOI:** 10.7759/cureus.32545

**Published:** 2022-12-15

**Authors:** Bithaiah Inyang, Feeba Sam Koshy, Kitty George, Prakar Poudel, Roopa Chalasani, Mastiyage R Goonathilake, Sara Waqar, Sheeba George, Wilford Jean-Baptiste, Amina Yusuf Ali, Lubna Mohammed

**Affiliations:** 1 Research, California Institute of Behavioral Neurosciences & Psychology , Fairfield , USA; 2 Research, California Institute of Behavioral Neurosciences & Psychology, Fairfield, USA; 3 Research, Chitwan Medical College of Medical Science, Chitwan, NPL; 4 Pediatrics/ Internal Medicine, California Institute of Behavioral Neurosciences & Psychology, Fairfield, USA; 5 Pediatrics, California Institute of Behavioral Neurosciences & Psychology, Fairfield, USA; 6 Internal Medicine, California Institute of Behavioral Neurosciences & Psychology, Fairfield, USA

**Keywords:** acalculous cholecystitis, lap chole, cholecyst, gallbladder, gallbladder disease, giant roundworm, ascaris worms, ascaris infection, ascaris lumbricoides, ascariasis

## Abstract

*Ascaris lumbricoides* is the most common type of helminth infection in humans. It affects more than one billion of the world's population. Children living in developing nations are prone to ascariasis, presenting with obstructive biliary illnesses. Migration of *Ascaris* worms through the major duodenal papilla to the hepatobiliary system leads to symptoms of biliary colic and complications along the biliary tree. In April 2022, we performed a systematic review of case reports to identify and examine cases of gallbladder ascariasis worldwide. A methodical search using PubMed, Semantic Scholar, ScienceDirect, and Directory of Open Access Journals yielded 2773 studies. After duplicate removal, title, abstract, and content screening, retrieval, and quality assessment, 13 studies met the criteria for this systematic review of case reports. The cases and results from these 13 studies revealed gallbladder ascariasis in different age groups worldwide. This systematic review discusses ascariasis, explicitly highlighting its presence in the gallbladder, symptomatic presentation, laboratory/imaging findings, complications, and approach to management.

## Introduction and background

Over one billion people worldwide have an *Ascaris* worm (AW) infection known as ascariasis. *Ascaris lumbricoides (A. lumbricoides*), the prominent human roundworm, is a parasitic nematode frequently found in endemic developing countries [1,2]. Ascariasis is prevalent in tropical areas with high-risk hygiene behaviors and inadequate sanitation [3]. Ascariasis is a leading cause of obstructive biliary disease in children living in developing countries with a high prevalence of infestation [4]. Children are most prone to ascariasis between ages three and eight [5]. Transmission is fecal-oral via ingestion of infective *Ascaris* eggs [2].

*A. lumbricoides*, the giant roundworm, is a soil-transmitted helminth; infection from this helminth occurs when an infected host deposits worm eggs in the soil during defecation. With adequate temperature and dampness, the worm eggs undergo modification to an infectious form awaiting infection of a human host via oral intake of eggs/contaminated foods [6].

Interestingly, fertilized eggs need at least 10 days to change into infectious parasitic eggs within the soil. Once ingestion of the eggs occurs, gastric acid within the stomach dissolves the protective eggshell leading to the birth of the embryo in the duodenum as a rod-shaped protoplasmic 13 to 15 μm thick and 200 to 300 μm long larva. The larva reaches sexual maturity in the small intestine, and an adult worm typically settles in the jejunum. *Ascaris* larvae are mobile and freely flow to the cecum, penetrating the mucosa and moving through the portal system to the liver, hepatic veins, heart, and lungs [7].

Most giant worm infections do not result in clinical symptoms, but clinical manifestation becomes almost inevitable in cases of heavy worm infestation. Several signs and symptoms are indicators of *A. lumbricoides* infestation; these include fever, vomiting, in some instances, live worms in vomitus, the passage of worms in stool, abdominal pain, abdominal right upper quadrant (RUQ) distension, jaundice, and hepatomegaly [4]. Ascariasis can also present with weight loss, lymphadenopathy, fatigue, and pulmonary symptoms (worms migrating through the tracheobronchial tree can cause irritation leading to pneumonitis) [7]. 

Although this review focuses on ascariasis in the gallbladder, it is vital to note that ascariasis can also infect other parts of the hepatobiliary system and the pancreas causing inflammation/infection and sometimes obstruction. Patients with a history of sphincterotomy or cholecystectomy presenting with biliary or pancreatic disease are at risk for ascariasis, especially in endemic countries [7]. Confirmation of diagnosis employs tools such as stool sampling for ova and parasites, radiographic imaging or sonography, and minimally invasive diagnostic techniques such as endoscopic retrograde cholangiopancreatography (ERCP) or endoscopy, which we will discuss more broadly in the discussion section [3,7]. This systematic review aims to review the presentation and management of gallbladder ascariasis (GA) by examining a broad patient demographic of cases worldwide.

## Review

Methods

In April 2022, we performed a systematic review of case reports to identify and examine cases of GA worldwide, strictly following the Preferred Reporting Items for Systematic Reviews and Meta-Analyses (PRISMA) [8]. We explored the following databases: PubMed, Semantic Scholar, ScienceDirect, and Directory of Open Access Journals (DOAJ). 

Search Strategy

On PubMed, we utilized the search strategy below by identifying keywords related to *A. lumbricoides* and the gallbladder; the final search strategy consists of these keywords and a medical subject headings (MeSH) combination built on Medical Literature Analysis and Retrieval System Online (MEDLINE) merged with Boolean operators as shown below: 

(Ascariasis OR *Ascaris Lumbricoides* OR *Ascaris* infection OR *Ascaris* infestation OR *Ascaris* worms OR Giant roundworm OR ( "Ascariasis/complications"[Majr] OR "Ascariasis/diagnosis"[Majr] OR "Ascariasis/drug therapy"[Majr] OR "Ascariasis/epidemiology"[Majr] OR "Ascariasis/etiology"[Majr] OR "Ascariasis/parasitology"[Majr] OR "Ascariasis/prevention and control"[Majr] OR "Ascariasis/therapy"[Majr] ) AND Gallbladder disease OR Gallbladder OR Cholecyst OR ("Gallbladder Diseases/parasitology"[Majr] OR "Gallbladder Diseases/pathology"[Majr] )

A combination of two crucial keywords, "Ascariasis AND Gallbladder disease", was employed on Semantic Scholar, ScienceDirect, and DOAJ to ensure specified results in our study area. 

Inclusion and Exclusion Criteria

During data extraction, we implemented the following criteria in Table 1 to sufficiently explore relevant cases related to GA and current management. 

**Table 1:** List of Inclusion and Exclusion Criteria

Criteria	Specification of Criteria
Criteria One	Fields of study relating to medicine.
Criteria Two	Review of reports with a publication date between the past 10 years (2012 - 2022).
Criteria Three	Selection of only case reports.
Criteria Four	Sorting particularly for free full-text articles.

Results

The search yielded 2773 publications from databases using the methods noted above. With the aid of EndNote (Clarivate, London, United Kingdom) and manual comparison, we identified 354 duplicates from the initial investigation. 2419 proceeded to undergo thorough title and abstract screening for applicability to our research topic. A total of 26 studies met the relevance screening of titles and abstracts, four of which could not be retrieved. We assessed the remaining 22 studies for specific relevance to the gallbladder disease process and quality using the Joanna Briggs Institute Critical Appraisal Checklist for Case Reports. Seven studies discussed biliary ascariasis without gallbladder involvement leading to exclusion, and we were unable to obtain two studies after multiple attempts to contact the authors for the full text.

**Figure 1:**
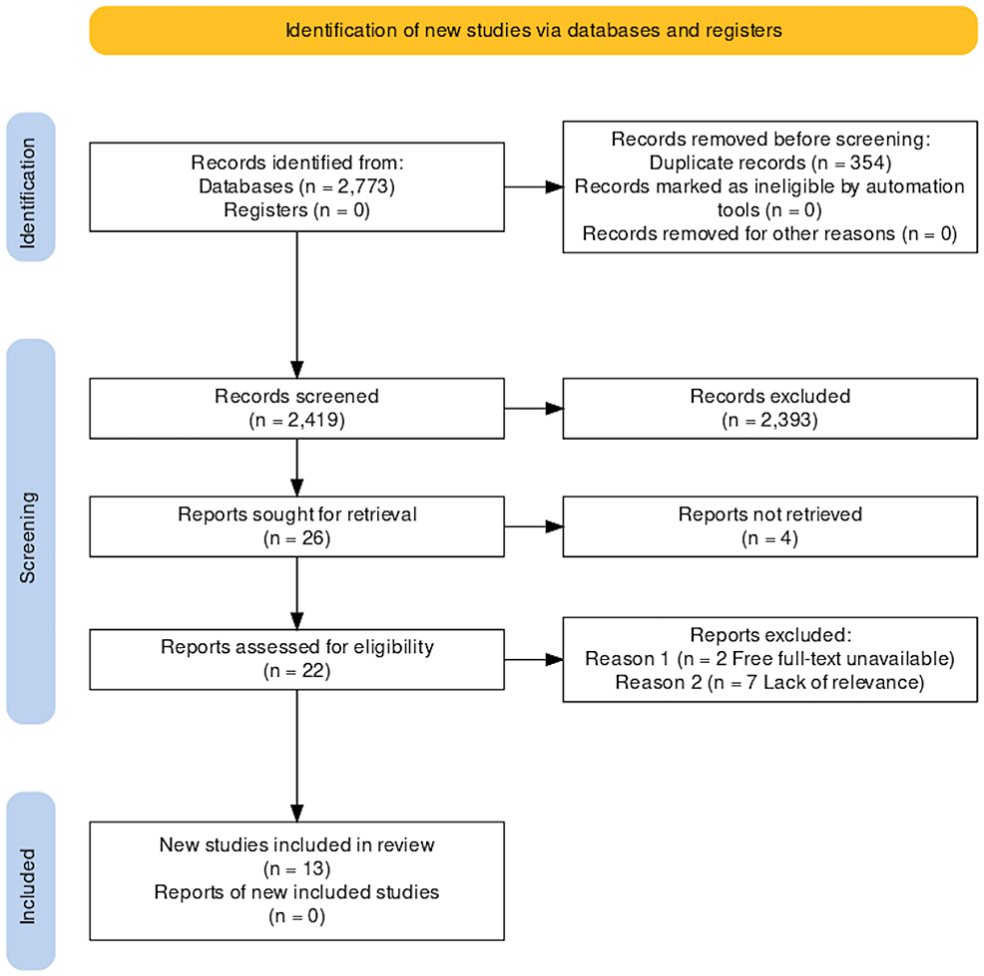
PRISMA 2020 Flowchart PRISMA: Preferred Reporting Items for Systematic Reviews and Meta-Analyses

Finally, we identified 13 studies to meet all our requirements. Figure 1 shows a breakdown of the results using a PRISMA flow diagram.

Table 2 includes a snapshot of the 13 studies highlighting the patient demographic, country of findings, patient presentation, and management of cases we will discuss further in the discussion section of this systematic review.

**Table 2:** Overview of Selected Cases IV: Intravenous, NPO: Nil per Os (Nothing by Mouth), RUQ: right upper quadrant, BID: Bis in Die (twice a day), ERCP: endoscopic retrograde cholangiopancreatography, T-tube: a T-shaped tube, and mg – milligram

Case Report Title	Author	Year	Patient Demographics	Country	Presentation	Management
Traditional herbal medicine therapy of gallbladder ascariasis: a case report.	Israyil et al. [1].	2021	Four-year-old; male; Uyghur native	China	Patient with a history of ascariasis with intermittent upper abdominal pain for seven days and subsequent yellowing of the sclera for three days.	Conservative treatment with Uyghur herbal preparations combined with magnesium sulfate for 10 days.
Successful elimination of gallbladder ascariasis by conservative therapy, followed by cholecystectomy due to developing cholecystitis	Alhamid et al. [9].	2018	Seventy-year-old; male	Syria	Incidental finding on ultrasonography.	Conservative treatment with two doses of albendazole 400 mg and empiric antibiotics, and eventually emergent cholecystectomy to avoid obstruction by worm debris.
Successful conservative management of uncomplicated gallbladder ascariasis	Thapa et al. [10].	2021	Sixteen-year-old; male	Nepal	Five-day history of intermittent epigastric pain and two episodes of vomiting (non-bilious).	Medical management with 400 mg of albendazole, IV fluid, ondansetron, and pantoprazole.
Not everything in the gallbladder is gallstones: an unusual case of biliary ascariasis	Keshvala and Naidu [5].	2019	Twenty five-year-old; female; Indian origin	Britain	Two-week history of fever and sore throat after treatment with amoxicillin and Augmentin. Presenting with nausea, vomiting, yellowish tinting of urine, and acute epigastric pain.	Mebendazole for three days.
Hepatobiliary ascariasis complicated by pancreatitis	Azhar et al. [11].	2015	Fifty two-year-old; Female	Pakistan	Three-day history of epigastric pain with associating nausea and bilious vomiting (“snake-like creature” found in vomitus).	Conservative management with empiric antibiotics, mebendazole 100mg 12 hourly, and NPO. Subsequent laparoscopic cholecystectomy for Ascaris lumbricoides extraction.
Gallbladder perforation due to Ascaris lumbricoides in a pregnant woman and 6 year old girl from Afghanistan: case report	Mosawi et al. [12].	2019	Case 1: Seventeen-year-old; female; pregnant Case 2: Six-year-old; female	Afghanistan	Case 1: Eight-day history of RUQ abdominal pain with development of anorexia, nausea, and vomiting one day before her presentation. Case 2: Three-day history of abdominal distension, pain, and vomiting.	Case 1: Common bile duct exploration and cholecystectomy with Ascaris worm removal. Case 2: Albendazole for biliary ascariasis and enterotomy with milking to remove worms
Gallbladder ascariasis with uneventful worm migration back to the duodenum: A case report	Mushtaque et al. [13].	2012	Forty-year-old; female	India	Patient with a history of acute acalculous cholecystitis presenting with live gallbladder Ascaris on ultrasound.	Cholecystectomy.
Gallbladder ascariasis in Kosovo – focus on ultrasound and conservative therapy: a case series	Ismaili-Jaha et al. [14].	2018	Case 1: Sixteen-month-old; female; Albanian Case 2: Twenty two-month-old; female; Albanian Case 3: Four-year-old; female; Albanian Case 4: Ten-year-old; male; Albanian	Kosovo	Case 1: Presenting with fever, diarrhea, and vomiting. Case 2: Presenting with weakness, fever, dehydration, diarrhea, and vomiting. Case 3: Presenting with vomiting, diarrhea, and fever. Case 4: Presenting with episodes of diarrhea and vomiting with fever.	Case 1: Mebendazole 100 mg BID for three days and antibiotics. Case 2: Mebendazole 100 mg BID for three days and antibiotics. Case 3: Mebendazole 100 mg BID for three days and antibiotics. Case 4: Mebendazole 100 mg BID for three days and antibiotics.
Gall bladder ascariasis: a rare entity	Gyawali et al. [15].	2021	Eight-year-old; female	Nepal	Two-day history of RUQ abdominal pain and non-bilious vomiting.	Supportive management and albendazole 400 mg. Preventive deworming with albendazole every six months.
Endoscopic ultrasound appearance of dead Ascaris lumbricoides in the biliary tract	Somani et al. [16].	2017	Two-year-old; female	India	Three weeks history of symptoms linked with biliary colic and obstructive jaundice.	ERCP and deworming with albendazole.
Combination of laparoscope and choledochoscope to treat biliary ascariasis	Cai et al [17].	2017	Sixteen-year-old; female	China	Ten-day history of RUQ abdominal pain with vomiting episodes (vomitus - four Ascaris-like worms).	Laparoscopic exploration and removal of worms from the biliary system with the placement of a T-tube. Three days of oral anthelmintics and daily T-tube rinse.
Biliary ascariasis and severe bacterial outcomes: report of three cases from a paediatric hospital in Brazil	de Almeida et al. [18].	2020	Case 1: One-year-old; male. Case 2: Three-year-old; male. Case 3: Seven-year-old; male	Bahia-Brazil	Case 1: Four-day history of fever, abdominal pain, distension, and diarrhea with one episode of oral expulsion of ascarids. Case 2: Four-day history of abdominal pain, fever, jaundice, constipation, choluria, and loss of appetite. Case 3: Three-day history of abdominal pain, distension, fever, loss of appetite, and jaundice.	Case 1: Managed conservatively initially with mebendazole, piperazine, metronidazole, and ceftriaxone. Due to worsening condition, further management with exploratory laparotomy with worm removal and a Kehr’s Tube placement. Case 2: Initial management with mebendazole and piperazine and then metronidazole and ceftriaxone for four weeks. Case 3: Conservative management with mebendazole, piperazine, metronidazole, and ceftriaxone.
Ascariasis in common bile duct resulting in a subhepatic abscess	Anzali et al. [19].	2020	Seventy-year-old; female	Iran	Two-week history of RUQ abdominal pain, weight loss, and episodic vomiting.	Conservative management with mebendazole 500 mg BID for three days, ciprofloxacin 400 mg BID, and metronidazole 500 mg every eight hours a week.

Discussion

*A. lumbricoides* typically presents in the gall bladder as acute acalculous cholecystitis. *A. lumbricoides* is the most common helminth; patients can have repeat occurrences of ascariasis disease [1,15]. Some countries (endemic regions in the Middle East, Far East, India, Latin America, and Africa) are more prone due to low socioeconomic status and lack of awareness about appropriate sanitary practices [12,13]. 

Life Cycle 

**Figure 2:**
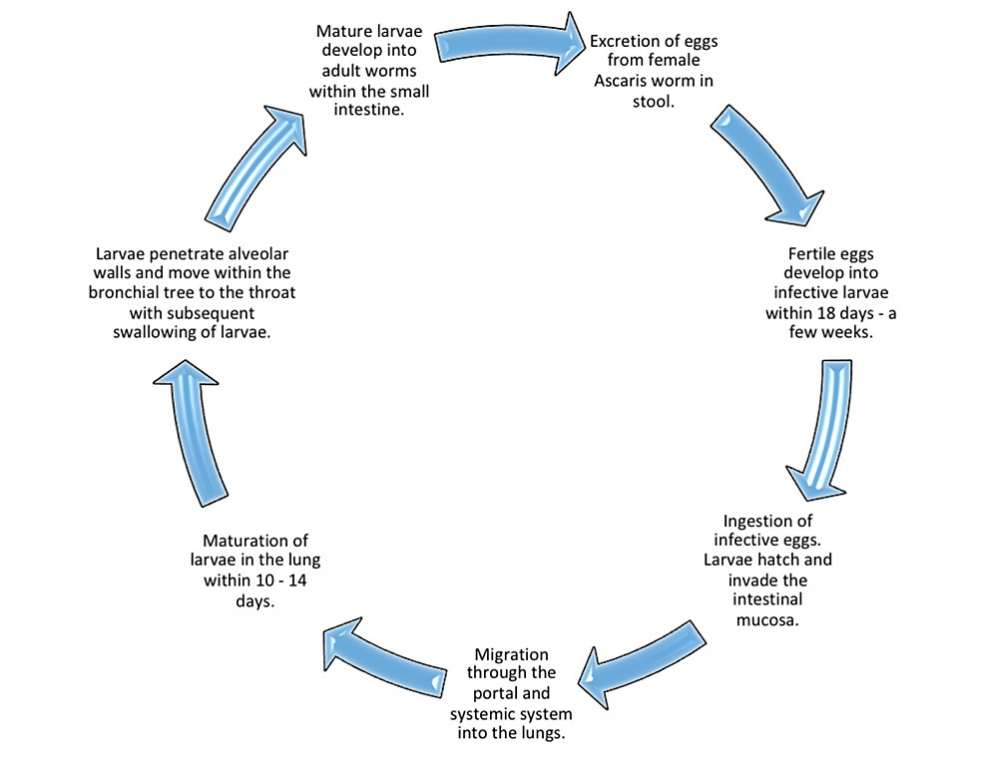
Life Cycle of Ascaris lumbricoides (Full Cycle Takes Two to Three Months) Figure Created by Author

It takes ~ two weeks from ingestion for the egg to reach the small intestine through the esophagus, respiratory tract, and liver. Adult male worms, 15-30 cm in length, and females, 20-35 cm in length, can survive in the intestines for one to two years [5]. Figure 2 illustrates the life cycle of A. lumbricoides [20].

*A. lumbricoides* infections are highly asymptomatic, and an adult AW can stay dormant in the lumen of the small intestine for a year or more. Small bowel obstruction is a common finding in infected human hosts [1,13]. Partial or complete intestinal obstruction is the primary clinical manifestation associated with pediatric hospitalization for ascariasis [18]. When the condition in the small intestine becomes less than ideal, the AW seeks to migrate to alternate organs, and the major duodenal papilla serves as a passage system from the duodenum to the hepatobiliary system through to the biliary system [17].

It is rare for the AW to invade the gall bladder because the biliary tract is fragile, narrow, and tortuous; only 2.1% of hepatobiliary ascariasis comes from the gallbladder [1,9]. The anatomical structure of the cystic duct makes it tedious for the AW to migrate to the gallbladder when there is a low count of worm infestation. A high volume load of AWs in the intestine can influence movement through the narrow cystic duct to the gallbladder through the ampulla orifice from the duodenum [10,11,13,18]. Worms tend to migrate when there is a load of 1000 AW or more from the intestine [13]. When worms and their eggs migrate to the common bile duct and cystic duct, it can obstruct the gallbladder, causing distension and acute cholecystitis [13,14].

Mushtaque et al. elaborated on a case where the AW migrated to the gallbladder and returned to the duodenum [13]. Factors that determine the migration of parasites to the gallbladder include the sex, age of the host, the amount of worm in the gut, and the worm's size [12,14]. A female AW is more likely to enter the hepatobiliary system [19].

Presentation

GA can be asymptomatic or present with gastrointestinal symptoms, severe infectious manifestations, and complications [9,14,18]. When the AW migrates out of the biliary tree, these symptoms worsen pain, fever, and jaundice [15]. Findings such as dyspepsia, anorexia, hepatomegaly, and vomiting of live AWs are clues to diagnosis [11,17,18].

**Figure 3:**
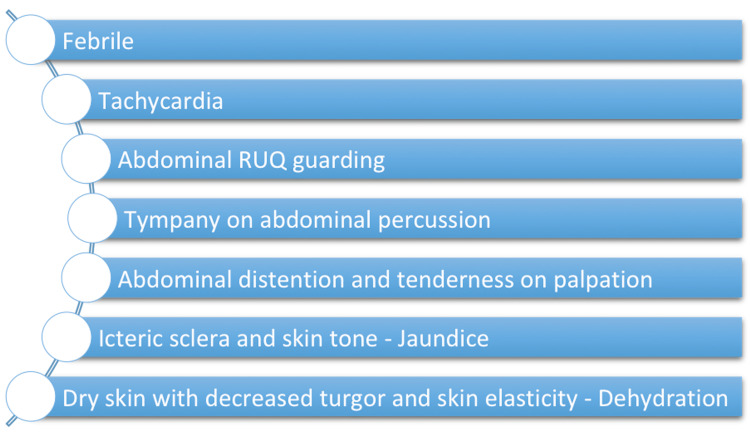
Physical Examination Findings in Gallbladder Ascariasis RUQ: Right upper quadrant Figure Created by Author

Figure 3 highlights the most common physical examination findings characteristic of GA. In countries with AW predominance, it is crucial to consider ascariasis in a child presenting with cholecystitis and obstructive jaundice [16,18].

Laboratory Findings 

Stool ova/parasite sampling is practical in areas with extreme prevalence of ascariasis. Stool/ova test revealing 60 x 50 um large, brown three-layered eggs is characteristic of ascariasis [11,14]. Male AWs can lead to false-negative studies in stool sampling for AW eggs [5].

Table 3 displays the laboratory findings notable in GA, with the prevalence of these findings. Abnormal elevated laboratory findings often tend to be normal within weeks to months of treatment.

**Table 3:** Laboratory Findings with Overall Incidence Percentage Noted in the Cases Discussed in Table 1

Laboratory Findings	Percentage of Cases with Findings
Leukocytosis	65%
Elevated liver enzymes	60%
C-reactive protein	35%
Ova and parasite test	35%
Elevated serum bilirubin	29%
Eosinophilia	29%
Erythrocyte sedimentation rate	18%
Amylase	12%
Lipase	6%
Leukopenia	6%
High serum and urine glucose	6%
Urine acetone and protein	6%
Positive smooth muscle antibody	6%
Serum total bile acid	6%
Cholinesterase	6%
Adenosine deaminase	6%

Imaging Diagnostic Studies

The following imaging studies are essential in diagnosing GA: ultrasonography, ERCP, abdominal magnetic resonance cholangiopancreatography (MRCP), and magnetic resonance imaging (MRI) [9].

X-ray abdomen: Non-specific GA, notable for cases with intestinal obstruction. Findings may include railway appearance, dilated small bowel loops with multiple air-fluid levels, and dark looped mass in the duodenum [12,18].

**Figure 4:**
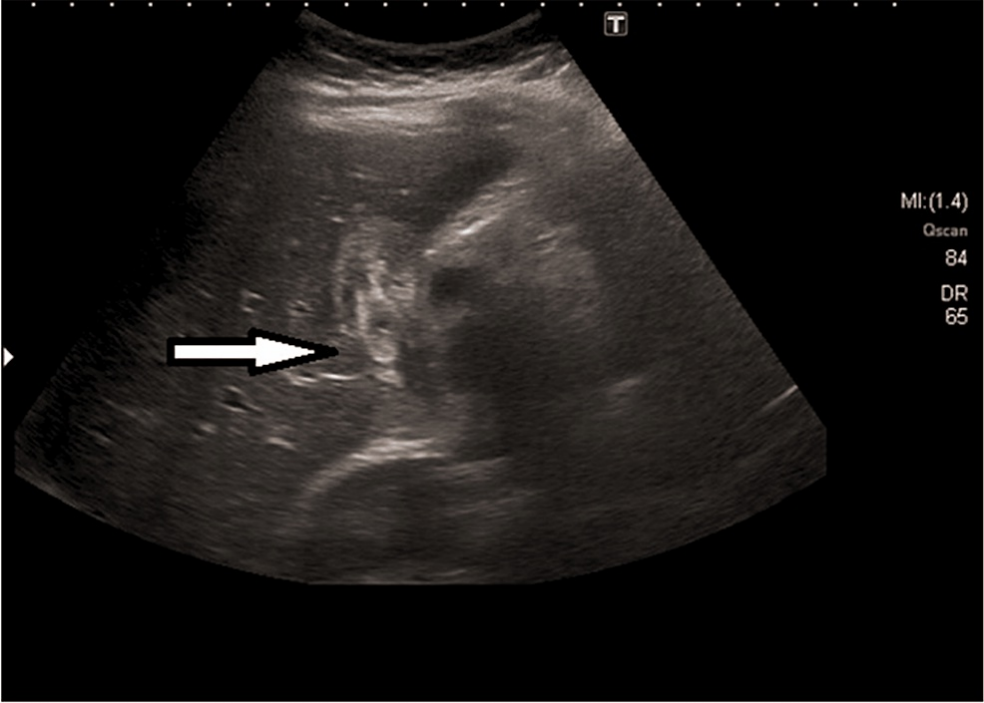
Ultrasound of a Gallbladder with Ascariasis Reproduced from Keshvala C, Naidu L: Not everything in the gallbladder is gallstones: an unusual case of biliary ascariasis. BJR Case Rep. 2019 [5] with permission from Keshvala C. The arrow highlights an Ascaris worm within a gallbladder with a classic bull's eye presentation. MI: Mechanical index; DR: dynamic range

Abdominal ultrasound (US):* *Ultrasonography is a standard non-invasive study for diagnosing gallstones; this diagnostic method effectively identifies biliary ascariasis. The AW shows up in imaging studies as a curved/linear moving echogenic structure with an anechoic central line and characteristic movement of these long echogenic structures within the bile duct. The AW localized within the gallbladder can present as an echogenic-filing defect appearing as a bull's eye [1,5,9-11,13-15]. Figure 4 shows the AW in the gallbladder presenting with a bull's eye appearance. 

The advantage of US to computerized tomography (CT) scan or MRI studies in biliary ascariasis is that it quickly reveals the worm's movement in the biliary tree [1]. US is preferable to MRCP and ERCP because of easy accessibility, cost, and safety. It has high sensitivity (85.7%) and is non-invasive [10,18]. US is the gold standard for GA because it is specific and safe. Roundworms always appear as echogenic structures, typically with an anechoic tubular center in a coil or strip. The movement of AW in the gallbladder is non-directional/not concrete. Findings include gallbladder distention with dilation of the common bile duct (CBD) and intrahepatic biliary radicles, gallbladder wall edema, sludge in the gallbladder and peri-cholecystic collection. Although the US can track AWs through the biliary tree, the middle and distal CBD can be difficult to visualize sometimes because of intestinal gas [15-17,19].

ERCP: US is safe and affordable but can miss 50% of infections in the duodenum and ampulla. However, ERCP can localize these structures. AW shows up as a tube-like, rounded, or linear filling defect on ERCP. ERCP is essential for removing AWs and exploring pancreatic ducts, CBD, and intestinal lumen [5,18,19].

**Figure 5:**
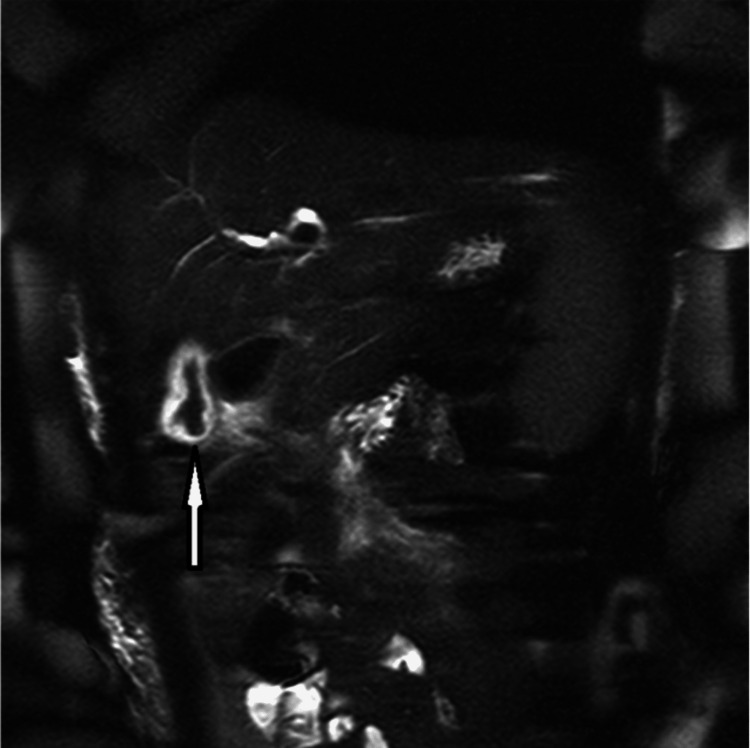
Abdominal Magnetic Resonance Cholangiopancreatography with Appearance of Ascaris worms in the Gallbladder Reproduced from Keshvala C, Naidu L: Not everything in the gallbladder is gallstones: an unusual case of biliary ascariasis. BJR Case Rep. 2019 [5] with permission from Keshvala C. The arrow localizes the gallbladder calling attention to the presence of Ascaris lumbricoides roundworms inside the distended gallbladder.

Abdominal MRCP: *A. lumbricoides* is constantly moving and can be challenging to pinpoint during imaging studies. MRCP can assist with localizing worms and inflammation in other areas of the hepatobiliary system. Intraductal worms show up as linear hypo-intense filling defects or bans with linear signals along the gallbladder wall. MRCP can confirm the findings in the gallbladder and pancreas head as AW [1,5,11,16]. Figure 5 shows MRCP of the abdomen with visible AWs in the gallbladder. 

MRI: Similar to MRCP, MRI is a three-dimensional imaging study. The AW shows up as a linear, slightly hyperintense tube-like structure with a hypointense central area. The disadvantage of MRI is the cost [15].

**Figure 6:**
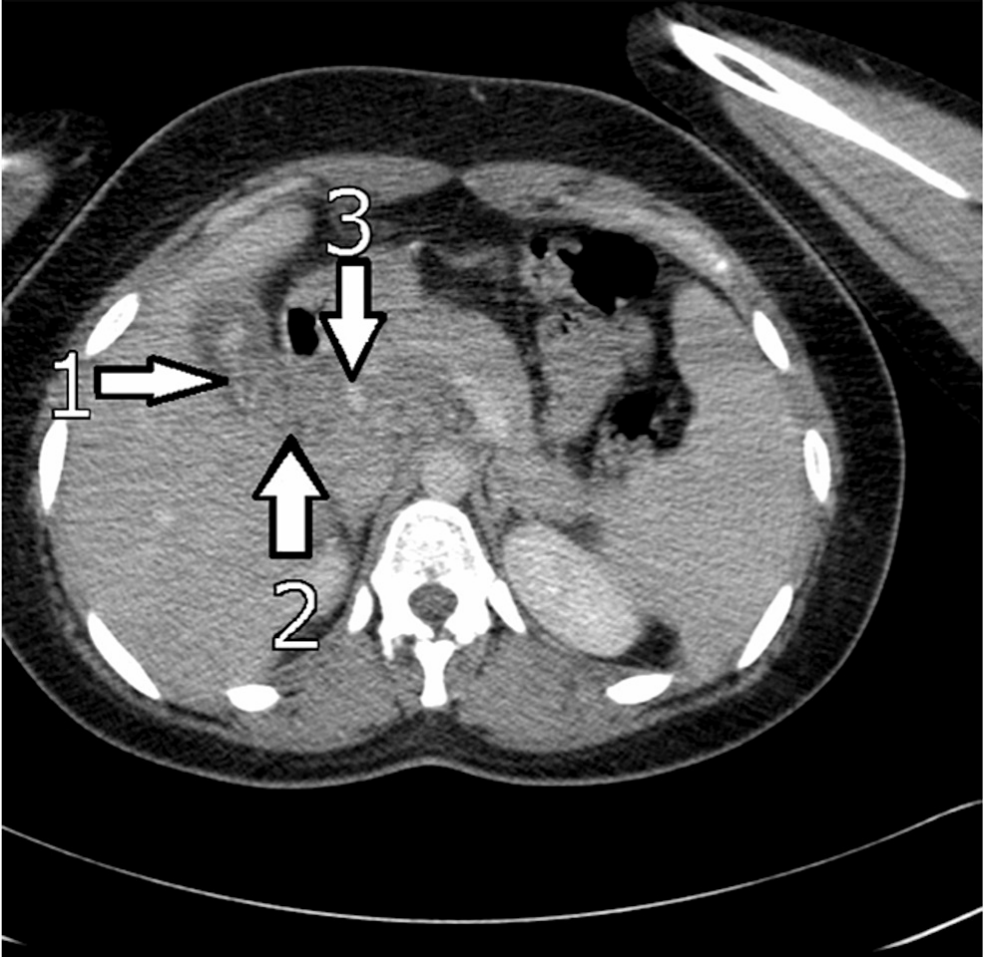
Abdominal Computerized Tomography Scan of a Gallbladder with Ascariasis Reproduced from Keshvala C, Naidu L: Not everything in the gallbladder is gallstones: an unusual case of biliary ascariasis. BJR Case Rep. 2019 [5] with permission from Keshvala C. Arrow 1: Ascaris worm detected within the gallbladder. Arrow 2: Highlights the common bile duct with Ascaris worm extending into the duct from the gallbladder. Arrow 3: Localizes the pancreatic head with surrounding edema.

CT scan: CT scanning is critical in spotting worms as AWs are visible on abdominal CT [5]. Findings include linear, curved solids in the gallbladder to the CBD and pancreas with distension of the uncinate process. CT is excellent at identifying complications like a hepatic abscess (A. lumbricoides in the biliary system is responsible for 14.5% of all liver abscess cases) and localizing inflammation like pancreatitis (high density) [5,18]. Figure 6 is an abdominal CT scan of a patient with GA showing the AW presenting with high intensity signals in the gallbladder and CBD. 

Pathologic/ histological findings: In some instances, pathology report of excise gallbladder reveals no AW, only an increase in eosinophils in the gall bladder's bile. Histological examinations can reveal focal infiltrations by lymphocytes reflective of chronic cholecystitis [9,11]. During imaging studies, AWs can mimic the following: Gallstones: echogenic foci on US; recurrent pyogenic cholangitis: duct dilation from intraductal stones; bacterial cholangitis: low-intensity signal in the ducts from purulent discharge; and cholangiocarcinoma: polypoid mass [5].

Complications

As discussed above, the AW lives in the jejunum; due to their mobile nature, worms are able to move to the biliary system through the papillary office leading to obstruction of the biliary system. Biliary obstruction is the cause of 10 - 19% of ascariasis hospitalization. AW migration to the gallbladder/biliary system can result in certain complications such as cholangitis, strictures, calculi, cholecystitis, pancreatitis, biliary colic, obstructive jaundice, ascending cholangitis, acute acalculous cholecystitis, pericholecystic abscess, liver abscess, pancreatitis, empyema, and sepsis [1,5,13,15,18]. 

Leftover bile duct/gallbladder worms can lead to a severe inflammatory response, decreased platelet count, and acute renal failure suspicious of sepsis that is often responsive to vitamin K and platelet transfusion. Other complications include stenosis, fibrosis, ductal necrosis, and calcification. Dead AWs within the biliary ducts can lead to strictures and severe inflammation. Worms in the CBD and cystic duct predispose patients to colic and cholecystitis [11,18,19]. 

AWs perforating the hepatic duct result in biliary peritonitis. *A. lumbricoides* in the biliary system is responsible for 14.5% of all liver abscess cases. Dead AWs can become a place for hepatic stone production in the CBD; live AWs produce glucuronidase, which can deconjugate bilirubin and result in precipitation and the formation of pigment stones. Hepatic abscesses could either be due to ascariasis or ERCP complications. Management of hepatic abscess is surgical with US-guided percutaneous drainage in addition to conservative therapy [17-19]. 

High parasite infestation leads to worm aggregation in the intestine leading to subsequent bowel obstruction, perforation, intussusception, or volvulus [5,12,19]. The AW migrating from the intestines to the biliary tree can carry bacteria from the intestinal wall to other organs causing complications of sepsis and hepatic abscess, which increases the necessity of broad-spectrum antibiotics, especially in cases with positive inflammatory signs [18,19].

Less than 2 % of biliary ascariasis cases have resulted in death despite complications [18]. The case management by Israyil et al. involving a combination of Urghur herbal preparations and magnesium had zero adverse effects [1].

Approach to Management

*A. lumbricoides* migrating to the gallbladder is a distinct occurrence that requires specialized treatment methods to eradicate. Initial management is conservative treatment with a vermifuge. Endoscopic and open surgical techniques are most appropriate for cases that fail conservative management. Treatment is started by keeping the patient nil per oral, and then fluid resuscitation is performed with intravenous fluids, antibiotics (if associated with infection/inflammation), and antispasmodics. Conservative treatment leads to the complete resolution of symptoms in 68 - 80% of individuals. Treatment with a vermifuge/anthelmintics such as albendazole, mebendazole, and pyrantel pamoate is crucial in the presence of live worms and most preferable after the worm is out of the biliary tract to prevent worm death and debris accumulation within the biliary tree [1,14,15].

Anthelmintics of choice include albendazole 400mg as a single dose, mebendazole 500 mg as a single dose, or mebendazole 100 mg twice daily for three days. 

Other anthelmintic drugs such as ivermectin, levamisole, and pyrantel pamoate can work for GA. If there is a whipworm co-infection, treatment of choice should be mebendazole. For patients requiring a water-soluble alternative with tube feedings, AW is treated with levamisole and pyrantel pamoate [5,10,14].

Anthelmintic medications function as paralytic agents for giant roundworms in the intestines and prevent the death of parasites within ducts [19]. In cases of GA with dead AWs, the goal is the elimination of worms killed in the gallbladder. Israyil et al. described the achievement of fecal elimination of AWs through gallbladder contraction; the four-year-old Uyghur boy underwent treatment with magnesium sulfate combined with Uyghur herbal preparation (Sirkenjibin buzur, Dinar sherbiti, and Kasini jewhiri). The herbal therapies promote gallbladder contraction, improving AW elimination while protecting the liver. Magnesium sulfate is cholagogic; it relaxes the sphincter of Oddi and surrounding muscles promoting evacuation of AWs from the gallbladder and bile duct into the duodenum for stool excretion [1]. 

Of note, less than 1% of anthelmintic medications undergo bile excretion, which is why GA can fail to respond to anthelmintic drugs until the AW migrates back to the small intestine, where the drugs can adequately target the worms. After treatment with anthelmintic agents, some patients eliminate roundworms through defecation. Utilizing a second course of anthelmintic can eliminate live worms present in imaging studies after initial vermifuge therapy. Within days of treatment, ascariasis completely resolves, and laboratory and imaging studies are back to normal with conservative management after four weeks [1,5,9,14].

As noted above, the treatment of GA heavily depends on presentation and complications. Management of simple cases involves conservative medication management, whereas complicated circumstances warrant a surgical or endoscopic approach to management. Escalation to surgical or endoscopic management is crucial if conservative management fails to yield positive results. Surgical management is considered for cases with a dead worm inside the gallbladder, gallbladder with both stones and worms, and lack of spontaneous expulsion of AWs from the gallbladder. Endoscopic removal of worms in CBD has proved successful in 90% of patients by using a balloon catheter or sphincterotomy to enable worm removal [5,10,14,19].

Endoscopic intervention is initiated when the AW is persistent within ducts for more than three weeks with no response to therapy, in cases with dead worms, and with recurrent cases. Management with endoscopic sphincterotomy is avoided since it creates a more accessible pathway for AW reentry [15]. Escalation to surgery is done if conservative and endoscopic treatment is unsuccessful. Laparoscopic exploration management plan is to visualize and explore the gallbladder and CBD. The AW is harvested from the CBD via laparoscopy; chodedochoscopy is performed to find less apparent worms within the biliary system, the AW is removed, a T-tube (a T-shaped tube) is inserted in the biliary duct for lavage, laparoscopic cholecystectomy is performed as needed, a drainage tube is placed, and AWs are retrieved. Extraction using forceps and a dormia basket is possible [12,15,17].

After surgical management, using anthelmintic medications and rinsing the T-tube daily with saline to extract more worms through the washout improve positive outcomes. A laparoscope with a choledochoscope best manages biliary ascariasis, especially for those with an enormous worm burden in the CBD. Cholecystectomy is recommended on account of echogenic debris (dead AW) accumulation in the gallbladder and acute cholecystitis [9,12,17].

With jejunal obstruction, enterectomy and worm removal is an appropriate course of therapy. Mosawi et al. described 7.7 lbs of AW expressed from the intestine after enterotomy and milking plus albendazole with smooth patient recovery [12]. It is crucial to keep in mind that patients with previous surgical histories on the biliary tract like sphincterotomy and Roux-en-Y hepaticojejunostomy develop biliary ascariasis more readily [13].

**Figure 7:**
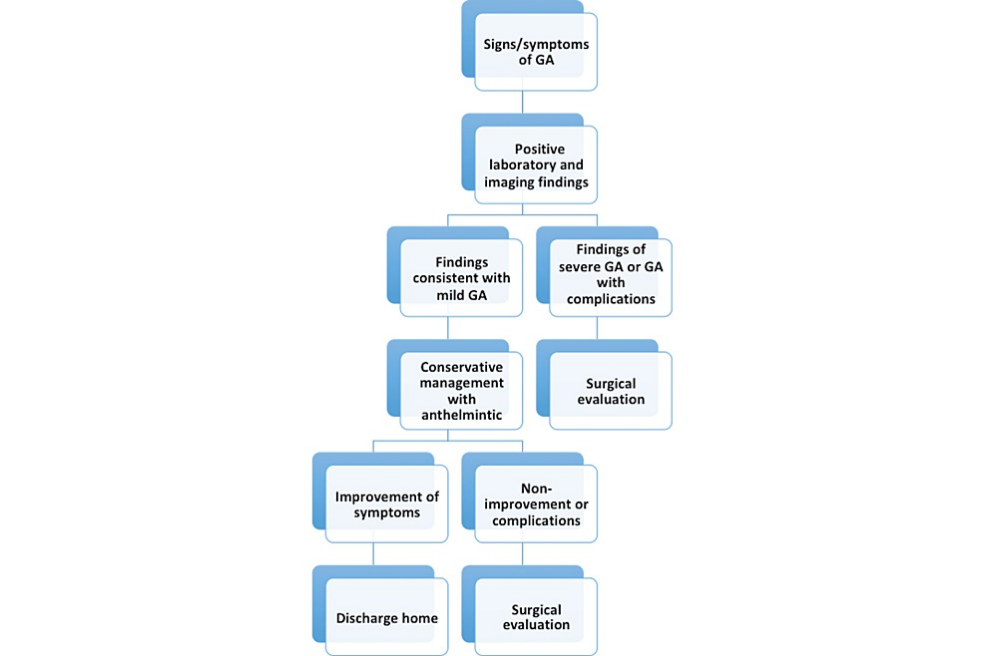
Summary of Approach to Management of Gallbladder Ascariasis GA: Gallbladder ascariasis Figure Created by Author

Recurrent worm invasion is typical; prophylactic deworming using albendazole for six months decreases the risk of recurrent ascariasis. Management of asymptomatic cases with deworming is crucial to prevent complications. In endemic countries, healthcare providers should encourage good sanitary practices such as washing hands and fruits/vegetables thoroughly or cooking raw fruits and vegetables before consumption. Educating patients on anthelmintic eradication and hygienic practices is integral to preventing ascariasis [10,13,15,19]. Figure 7 summarizes the recommended approach to management.

Pregnancy and Ascariasis

Ascariasis presents a remarkable risk with pregnancy. With each surgical procedure, there is a 21% mortality rate of ascariasis in pregnancy and a possibility of fetal demise. Risk in pregnancy increases due to hormonal levels of estrogen and progesterone affecting the sphincter of Oddi, facilitating the movement of AWs into the biliary duct and enabling the trapping of these worms [12].

US remains the safest imaging option in pregnancy. Contraindication exists with mebendazole and albendazole in pregnancy. In contrast, pyrantel pamoate and piperazine citrate treat ascariasis without risk of harm in late pregnancy [12].

Limitations

This review contains a few limitations. First, the intentional selection of studies published within the past 10 years and the systematic review of only case reports resulted in the elimination of relevant studies that failed to meet these inclusion criteria.

In addition, this review specifically focused on GA with a brief discussion of findings in other parts of the biliary system; this limitation implies that careful interpretation of the findings reported herein is necessary when considering ascariasis involvement in adjacent structures. Future studies exploring ascariasis involvement in different organs/systems would prove indispensable in managing cases involving giant roundworms.

## Conclusions

Although a rare presentation of *A. lumbricoides* infection, GA is a notable cause of biliary colic. This systematic review overviews adult ascariasis worms in the biliary system, especially in cases with high worm loads. Evidence indicates that children, patients living in endemic countries, and individuals with travel histories to these areas are especially at risk of developing cholecystitis and obstructive jaundice due to Ascaris worm infection. These findings should encourage healthcare professionals to emphasize the complete evaluation of patients in the at-risk group. Considering GA as a differential diagnosis in patients presenting with gastrointestinal or biliary symptoms in endemic regions is necessary to avoid delay in appropriate management. Pregnancy-related GA requires tremendous caution in treatment to prevent maternal and fetal loss. Present research findings have limitations in analyzing variables influencing the sphincter of Oddi other than pregnancy. Further expansion of these factors is integral in identifying the best management approaches in more specified cases of GA.
